# The Effects of Aphid Traits on Parasitoid Host Use and Specialist Advantage

**DOI:** 10.1371/journal.pone.0157674

**Published:** 2016-06-16

**Authors:** Vesna Gagic, Olivera Petrović-Obradović, Jochen Fründ, Nickolas G. Kavallieratos, Christos G. Athanassiou, Petr Starý, Željko Tomanović

**Affiliations:** 1 CSIRO, GPO Box 2583, Brisbane, QLD, 4001, Australia; 2 Institute of Zoology, Faculty of Biology, University of Belgrade, Belgrade, Serbia; 3 Faculty of Agriculture, University of Belgrade, Belgrade–Zemun, Serbia; 4 Department of Integrative Biology, University of Guelph, Guelph, Ontario, Canada; 5 Agrarökologie, Georg-August-Universität, Göttingen, Germany; 6 Laboratory of Agricultural Zoology and Entomology, Department of Crop Science, Agricultural University of Athens, Athens, Attica, Greece; 7 Laboratory of Agricultural Entomology, Department of Entomology and Agricultural Zoology, Benaki Phytopathological Institute, Attica, Greece; 8 Laboratory of Entomology and Agricultural Zoology, Department of Agriculture, Crop Production and Rural Environment, University of Thessaly, Magnissia, Greece; 9 Laboratory of Aphidology, Department of Experimental Ecology, Institute of Entomology, Biology Centre, Academy of Sciences of the Czech Republic, ČeskéBudějovice, Czech Republic; French National Institute for Agricultural Research (INRA), FRANCE

## Abstract

Specialization is a central concept in ecology and one of the fundamental properties of parasitoids. Highly specialized parasitoids tend to be more efficient in host-use compared to generalized parasitoids, presumably owing to the trade-off between host range and host-use efficiency. However, it remains unknown how parasitoid host specificity and host-use depends on host traits related to susceptibility to parasitoid attack. To address this question, we used data from a 13-year survey of interactions among 142 aphid and 75 parasitoid species in nine European countries. We found that only aphid traits related to local resource characteristics seem to influence the trade-off between host-range and efficiency: more specialized parasitoids had an apparent advantage (higher abundance on shared hosts) on aphids with sparse colonies, ant-attendance and without concealment, and this was more evident when host relatedness was included in calculation of parasitoid specificity. More traits influenced average assemblage specialization, which was highest in aphids that are monophagous, monoecious, large, highly mobile (easily drop from a plant), without myrmecophily, habitat specialists, inhabit non-agricultural habitats and have sparse colonies. Differences in aphid wax production did not influence parasitoid host specificity and host-use. Our study is the first step in identifying host traits important for aphid parasitoid host specificity and host-use and improves our understanding of bottom-up effects of aphid traits on aphid-parasitoid food web structure.

## Introduction

The trade-offs hypothesis suggests that specialization towards a host or a group of hosts is accompanied by an increase in host-use efficiency [1]. This has been documented for parasites [2, 3] and aphid parasitoids [4, 5] where abundance of parasitoids on their hosts was used as a proxy for host-use efficiency. Higher host-use efficiency allows specialists to coexist with generalists on the shared resources, but this “specialist advantage” may be context-dependent [6]. In the case of parasitoids, specialists may have an advantage and specialize towards hosts with certain traits [7, 5], but the relative importance of different host traits remains largely unknown. Identifying these traits can help identification of general patterns and hence make aphid-parasitoid interactions more predictive [8]. It improves our understanding of processes that govern parasitoid specialization and host-use and increases our knowledge that is currently based on a large number of species-specific choice experiments.

Host traits related to host defenses and host detection are likely to be important for koinobiont parasitoids and probably more so than species body size, the only trait commonly investigated in predator-prey food webs [9]. Specialization on certain hosts may improve parasitoids’ abilities to find suitable hosts or to overcome host defense mechanisms [10, 11]. More specialized parasitoids are often expected to be more efficient and thus more abundant on hosts that are less susceptible to parasitoid attacks [12]. For example, more specialized parasitoids capable of overcoming ant attacks due to e.g. chemical or behavioral adaptations may be more common and more efficient on ant-attended aphids. However, for some host traits it is hard to predict the direction of their effect due to multiple possible mechanisms affecting parasitoid host-use and specialization. A single trait, for example large aphid colony size, may simultaneously make a colony easy to locate even for generalists, but also increase the strength of aphid defense, thereby requiring some adaptation to overcome host defense mechanisms. To the best of our knowledge, our study is the first attempt to quantify the net effect of multiple aphid traits on parasitoid specialist advantage (but see [13, 14, 15] for the effect of individual aphid traits, such as presence of endosymbiotic bacteria or aphids feeding on toxic plants, on parasitoid host specificity).

Our understanding of the effects of aphid traits on parasitoid communities comes mostly from studies of parasitoid assemblage sizes, i.e. number of parasitoid species per aphid species [11, 16, 17, 18, 19, 20, 21]. These studies revealed some aphid traits, such as facultative ant-attendance, host plant-alteration, polyphagy, habitat disturbance (agricultural and urban vs. natural habitats) to be positively associated with aphid parasitoid assemblage sizes, while colony aggregation, host mobility, body size and wax production appeared to have no influence [18, 19]. However, less is known about the effects of these aphid traits on parasitoid host specificity and specialist advantage.

**Fig 1:**
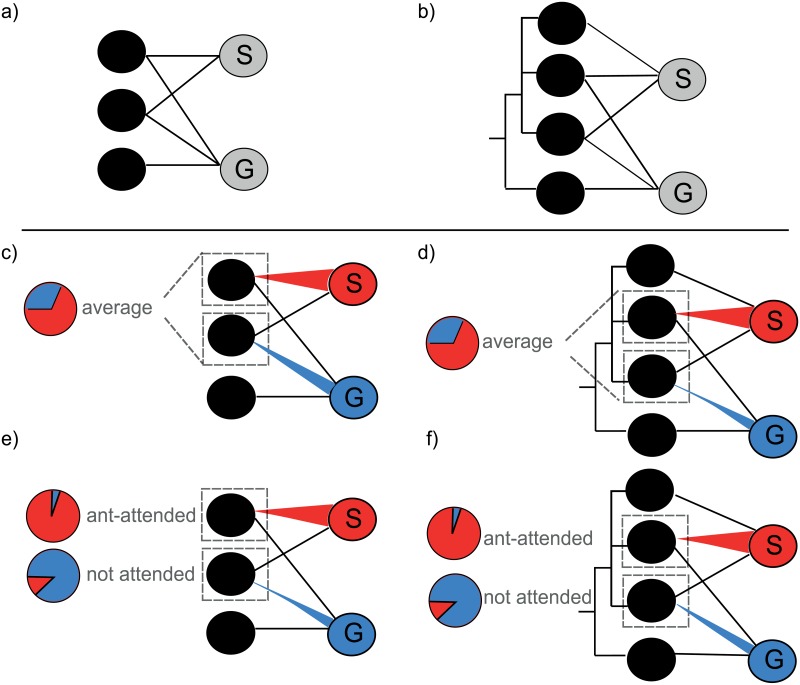
Specialist-generalist trade-off calculated using two measures of parasitoid specialization based on the number (a) and taxonomic diversity (b) of species in the host range. Upper panel illustrates the difference between two measures of host specialization and the lower panel illustrates theoretical expectations for our analysis. For pairs of parasitoids, the proportional abundance of the relative specialist (red, “S”) and generalist (blue, “G”) on the shared hosts is compared. Proportion of specialists can be measured across all shared hosts (c, d, see also [5]) or dependent on the host traits (e, f, our study). Black circles present different host species and the width of the red and blue links presents frequency of aphid-parasitoid interactions. Dashed squares highlight shared hosts, for which pie charts indicate the proportional abundance of relative specialists and generalists.

Host specificity is one of the fundamental properties of parasitoids and it influences the ability of parasitoids to use novel hosts and habitats, affects their vulnerability to extinction and determines their biocontrol potential [22, 23, 24]. Parasitoid specificity can be defined as the number (Fig 1a) or taxonomic diversity (Fig 1b) of species in the host range [25, 26]. Degree of host specialization may be phylogenetically conserved and aphid parasitoids are often restricted to develop in certain host species, genera or higher taxa [27]. Thus, an important limitation to parasitoid host ranges and the structure of aphid-parasitoid food webs is set by phylogenetic distance among hosts [28]. The cost of adaptation to phylogenetically more related hosts may be lower because closely related hosts are often more similar in ecological, behavioral, physiological or biochemical traits. Hence, even when two parasitoids attack the same number of host species, the one which attacks more related hosts can be considered as more specialized (Fig 1b). In plant-herbivore interactions, Rasmann & Agrawal [29] showed that both, host plant phylogenetic distance as well as host plant defense traits and habitat affiliation restricted herbivore host range. The trade-off between dietary breadth and host-use efficiency may result in higher overall abundances of specialists (“specialist advantage”) across all hosts they share with generalists (Fig 1c). This has been confirmed for aphid parasitoids, but only when the measure of specialization accounted for host relatedness (Fig 1d) [5]. However, specialists are likely to adapt to certain host characteristics, for example ant-attendance, while occasionally attacking other hosts. This should result in a higher specialist advantage on ant-attended compared to non-attended hosts (Fig 1e and 1f). Which aphid traits affect parasitoid host specificity and specialist-generalist trade-off and in which direction is still not well understood.

Aphid parasitoids represent a particularly interesting group for the analysis of specialist-generalist host-use for several reasons. First, parasitoids may comprise 20–25% of all insect species [11] and aphids (Aphidoidea) and their parasitoids (Aphidiinae) can be found in a large variety of habitats and in almost all climatic regions in the world. Second, aphid parasitoids are important agents in biological pest control and their host-use efficiency is therefore of particular interest [27, 30]. Finally, aphid parasitoid are a good model system because closely related parasitoids can differ in host specificity and a number of coexisting parasitoid species can exploit the same aphid host, allowing for comparative studies of parasitoid host specificity and host use. The lack of field studies on the relationship between aphid traits and parasitoid host specificity and specialist advantage is probably related to the fact that they require exhaustive large scale field surveys of a broad range of potential hosts and high expertise in plant, aphid and parasitoid taxonomy [11, 31]. On the other hand, existing experimental studies are species-specific and cannot be easily generalized to the trait level. Here, we identified the most important aphid traits influencing parasitoid host specificity and the generalist-specialist host use by analyzing data from a 13-year survey identifying plant-aphid-parasitoid links with high taxonomic resolution and across different habitats in nine European countries [32]. We aim to evaluate the importance of aphid traits on host specificity and specialist advantage, i.e. the relative abundance of specialist compared to generalist parasitoids on shared hosts. We test whether the power to detect influence of host traits depends on accounting for host relatedness in the measure of parasitoid specificity. We further examine the correlations among aphid traits and consider them for the interpretations. Our results will help to understand how the net advantage of specialization depends on host traits.

## Materials and Methods

### Data description

We analyzed data on aphid-parasitoid interactions originally reported by Kavallieratos et al. [32], based on a 13-year survey of parasitoids (Hymenoptera: Braconidae: Aphidiinae) and their hosts in southeastern Europe (Serbia, Montenegro, Greece, Bosnia, Croatia, The Former Yugoslav Republic of Macedonia, Bulgaria, Turkey and Cyprus). Plants bearing both live and mummified (parasitized) aphids were collected from many different localities and parasitoids were reared from the mummies in laboratory conditions (22.5°C, 65% relative humidity, 16: 8 L: D photoperiod). Hosts and parasitoids were identified to the species level. For the purpose of our analysis, poorly sampled interactions, i.e. those with fewer than five individuals, were excluded from the dataset. This is because these rare records represent low importance for parasitoid and host autecology and phylogeny and by excluding those data possible erroneous records are weeded out [26].

### Aphid traits

We examined the following aphid traits that we expected to influence the proportion of specialists on the shared hosts based on previous research: (a) Aphid concealment: 1. colony exposed on the open surface, 2. colony semi-concealed (inside curled leaves, galls or on roots); (b) Aphid colony aggregation: 1. dense, 2. sparse (individuals do not touch one another); (c) Ant-attendance (myrmecophily): 1. absent, 2. facultative, 3. obligatory; (d) Mobility (how quickly aphids drop from the plant when disturbed): 1. low, 2. high; (e) Wax production: 1. absent, 2. present; (f) Average adult body size: 1. large (3.5–9.0 mm), 2. medium (2.0–3.5 mm), 3. small (1.2–2.0 mm); (g) Aphid life cycle: 1. with host-plant alternation (heteroecious), 2.without host-plant alteration (monoecious); (h) Aphid diet breadth: 1. monophagous, 2. oligophagous (feeds on two or more plant species from same plant family), 3. polyphagous (feeds on two or more plant species from different plant families). (i) Habitat specialization: 1.habitat specialist (limited to one or several specific types of habitats) 2. habitat generalist (uses more types of habitats); (j) Habitat disturbance: 1. aphid found only in semi-natural habitats, 2. aphid found also in agricultural habitats. Aphid traits were found in Blackman and Eastop [33, 34, 35] and Heie [36, 37, 38, 39, 40, 41]. In several cases when literature data were not available the traits were assigned to aphids based on the authors’ personal observations during 13 years of sampling.

### Analysis

We calculated host range as: (1) number of host species from which a parasitoid emerged (SR—shown to be independent of parasitoid taxonomy in Straub, Ives & Gratton [5]) and (2) phylogenetic diversity of the hosts (PSV, [42]), modified for use with taxonomies [5]. PSV is robust to variation in sampling effort and species richness (for detailed explanations and calculations see Helmus et al. [42] and Straub, Ives & Gratton [5]). All analyses were performed in R version 3.1.0 [43].

#### Influence of host traits on the proportion of specialists on the shared host

Following Straub, Ives & Gratton [5] we looked at all pairwise comparisons between two parasitoid species that shared at least one host species and had different host ranges. The parasitoid species which had the smaller host range was then labeled “relative specialist” (hereafter “specialist”) and the species with the larger host range “relative generalist” (hereafter “generalist”). We measured host use efficiency as the abundance of parasitoids on their shared hosts [5, 44]. To explore the effect of aphid traits on parasitoid specialist advantage we performed an exploratory analysis for all traits by first separating data in subsets for each level of aphid trait. Second, for each subset we selected all hosts that were shared by the pair of parasitoids and calculated the mean relative parasitism of the relative specialist as the mean across all of the hosts it shared with the relative generalist. The mean relative parasitism of all specialists from all pairwise comparisons (mean score) was then calculated for each level of aphid trait. The pairwise comparisons avoid a confounding influence of sampling effort when calculating parasitoid host use efficiency. This is because in pairwise comparisons each parasitoid species in the pair has probability of emergence independent of aphid sampling intensity.

To determine whether there was a statistically reliable difference in the mean scores among aphids with particular traits we used a nonparametric bootstrap test (one test per trait). We computed Monte Carlo one-tailed p-values by drawing 4999 bootstrap replicates from the data. More specifically, we generated a bootstrap replicate by sampling rows (aphid species) in the aphid-parasitoid matrix with replacement (maintaining the number of species per trait category) and calculated a difference among “mean scores” (as described above) that we compared to the observed value. The p-values were calculated by counting the number of cases the simulated difference in mean scores is greater/lower from the observed difference and dividing by the number of bootstrap replicates

#### Influence of host traits on the average weighted specialization of parasitoid assemblage

To investigate the effects of aphid traits on the average weighted specialization of parasitoid assemblage we first summed the product of specialization index (S_*i*_ = PSV or SR) of all the parasitoid species attacking an aphid species and their relative abundances (A_*i*_) on that aphid species: ∑(S_*i*_ x A_*i*_) where subscript *i* denotes aphid species (*i* = 1:142). We then calculated mean values for each aphid trait level and tested the difference in means by using nonparametric bootstrap test as described above.

#### Relationship among aphid traits

The influences of different aphid traits on parasitoid host use cannot be interpreted in isolation from one another. To test the relationships among categories of different aphid traits we performed Multiple Correspondence Analysis (MCA), which is a specific application of correspondence analysis (CA) with multiple categorical variables [45]. MCA can also be viewed as a PCA applied on categorical data where the categories are represented at the barycentre of the individuals with those categories [41]. Because principal dimensions of the MCA may be influenced by a few missing values in the data, we used the “imputeMCA” function from the “missMDA” package in R [46] which uses the regularized iterative MCA algorithm to impute missing values [47]. For testing relationship between trait levels among different aphid traits, we calculated the v.test following Husson, Lê & Pagès [45] and using the “catdes” function in the “FactoMineR” package [48]. This function calculates the proportion of species which possess one category of the factor among those that possess a category of another factor, hence the v.test may differ depending on which category is tested first (e.g. the proportion of semi-concealed among ant-attended is not the same as the proportion of ant-attended among semi-concealed). If the absolute value of the v.tes is greater than 2, the coordinate can be considered significantly different from 0.

Convergence and conservatism in defense traits, together with the knowledge about strength and specificity of trophic interactions, is important to evaluate the role of trophic interactions affecting community structure [49]. See S1 Table for the results of measuring phylogenetic signal in aphid traits.

## Results

In total, we analyzed 31465 aphid-parasitoid interactions containing 142 aphid and 75 parasitoid species (see S2 Table for the list of species, S3 Table for the list of aphid traits and S4 Table for the aphid taxonomic data).

### Influence of host traits on the proportion of specialists on the shared host

When species richness was used as measure of parasitoid host range, only one trait had a significant effect: specialists were more abundant than generalists on shared hosts that form sparser colonies compared to those with dense colonies (diff. in means = 0.30, p = 0.003). When specialists were defined as parasitoids that attack one or a few closely related hosts (low PSV), more traits had significant effects: specialist relative abundance was higher on hosts that are exposed compared to semi-concealed hosts (diff. in means = 0.20, p = 0.004), hosts that form sparser colonies compared to those with dense colonies (diff. in means = 0.10, p = 0.02) and hosts that have myrmecophily compared to those that are not ant-attended (diff. in means = 0.18, p = 0.002), with a higher proportion of specialist on hosts that have facultative ant-attendance compared to obligate (diff. in means = 0.18, p = 0.02).

### Influence of host traits on the average weighted specialization of parasitoid assemblage

**Fig 2:**
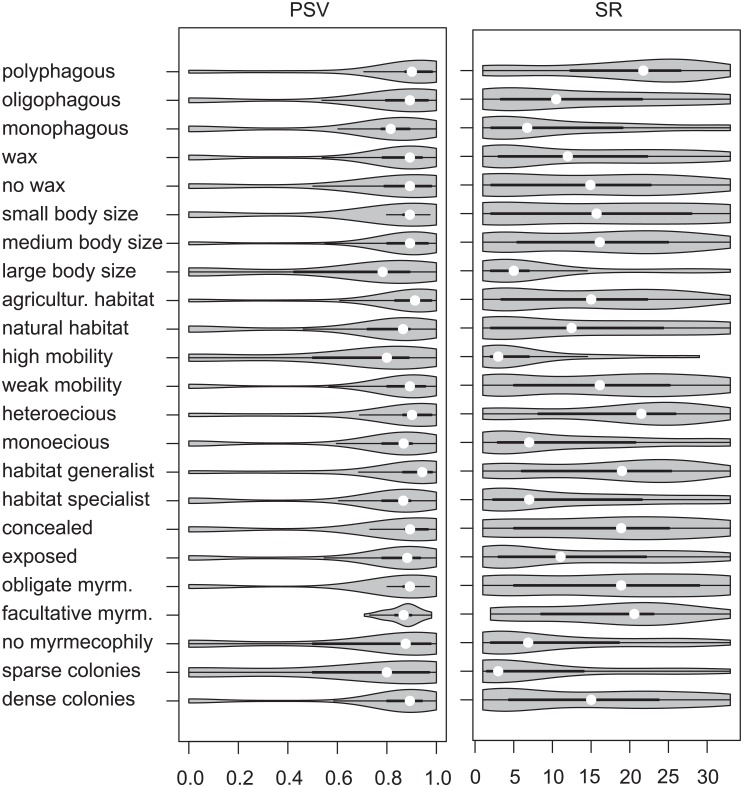
Violin plots presenting distribution of average weighted specialization of parasitoid assemblages per aphid trait. The median value for each aphid trait is shown by the white circle and the length of the thick black line represents the interquartile range. Parasitoid specialization is measured as species richness (SR) or phylogenetic diversity of the hosts (PSV).

Mean weighted parasitoid specialization was higher on the hosts that are habitat specialist compared to habitat generalist, have high compared to weak mobility and not ant-attended compared to ant attended, when using both indices for parasitoid specialization (Table 1, Fig 2). Aphids that have facultative ant attendance did not differ in mean specialization from those with obligate ant attendance. There was a gradient of decreasing mean parasitoids specialization from mono- to polyphagous aphids and from large to small aphid body sizes, where aphids with medium body size did not differ from those with small.

**Table 1:** Average weighted specialization of parasitoid assemblage per aphid trait level and corresponding p-values as a result of bootstrap test. Parasitoid specialization is measured as species richness (SR) or phylogenetic diversity of the hosts (PSV).

Aphid trait	Trait level	PSV	p-value	SR	p-value
Habitat specialization	Specialist	0.71	0.042	12.38	0.017
	Generalist	0.81		16.81	
Mobility	High	0.64	0.025	6.45	0.0001
	Weak	0.77		16.28	
Ant-attendance	Absent	0.69	0.01^a^	10.85	0.0004^a^
	Facultative	0.87		17.11	
	Obligate	0.78		17.33	
Diet breadth	Monophagous	0.70	0.028^b^	11.04	0.0002^b^
	Oligophagous	0.76		13.31	
	Polyphagous	0.81		18.65	
Body size	Large	0.64	0.032^c^	8.28	0.0002^c^
	Medium	0.80		15.99	
	Small	0.72		15.97	
Life cycle	Monoecious	0.72	> 0.1	12.18	0.0024
	Heteroecious	0.80		18.20	
Colony aggregation	Sparse	0.65	0.074	8.55	0.006
	Dense	0.76		14.90	
Habitat disturbance	Semi-natural	0.68	0.008	14.30	> 0.1
	Agricultural	0.82		14.08	
Concealment	Exposed	0.74	> 0.1	13.23	0.068
	Semi-concealed	0.76		16.80	
Wax production	Absent	0.75	> 0.1	14.14	> 0.1
	Present	0.74		13.76	

^a^—p-value presents difference between not ant-attended vs. ant-attended aphids (both facultative and obligate)

^b^—p-value presents difference between monophagous and polyhagous aphids

^c^—p-value presents difference between large vs. medium and small body sizes.

### Relationship among aphid traits

**Fig 3:**
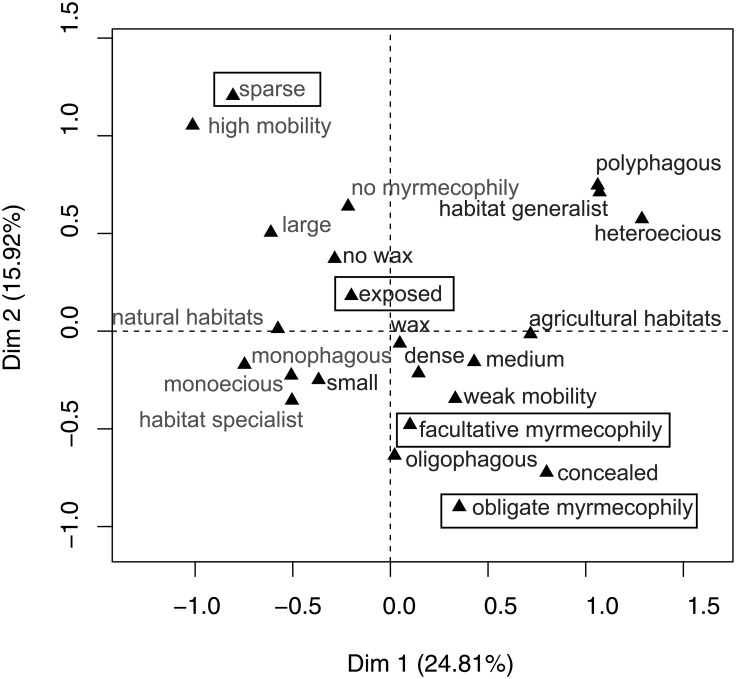
The first two principal axes (Dim1 and Dim2 with percentage of variance) and the cloud of categories as a result of MCA (Multiple correspondence analysis) on the aphid species × aphid traits matrix. Trait levels that are close together tend to be associated in aphids. Framed labels present trait levels which supported a higher proportion of relative specialists (specialist advantage). Gray labels present traits levels that supported higher average specialization of parasitoid assemblages. See the text for explanation of the traits and trait levels.

The effects of different aphid traits on parasitoid host use cannot be seen in isolation from one another, as these traits are often related (Fig 3). Aphid species with facultative ant-attendance were often characterized by weak mobility (v.test = 3.73, p = 0.0001), obligate ant-attended aphids were related to weak mobility (v.test = 4.52, p< 0.0001) and concealment (v.test = 4.89, p< 0.0001) and aphids that are not ant-attended often had high mobility (v.test = 6.75, p< 0.0001) and sparse colonies (v.test = 3.60, p = 0.0003). Aphids with high mobility were often not ant-attended (v.test = 6.75, p< 0.0001), had sparse colonies (v.test = 5.38, p< 0.0001), large body size (v.test = 4.55, p< 0.0001), inhabited natural habitats (v.test = 4.05, p< 0.0001), were exposed (v.test = 3.82, p< 0.0001) and monophagous (v.test = 3.37, p< 0.0001). Aphids that were restricted to semi-natural habitats were often habitat specialists (v.test = 5.70, p< 0.0001), monophagous (v.test = 5.62, p< 0.0001), monoecious (v.test = 4.79, p< 0.0001) with high mobility (v.test = 4.05, p< 0.0001).

## Discussion

In this study we show that relative specialist and generalist parasitoids differ in their host use efficiency and average specialization of assemblages across aphid hosts possessing certain traits. Not all host traits supported both, higher average parasitoid specialization and higher specialist advantage. Average assemblage specialization was the highest on aphids that are monophagous, monoecious, large, highly mobile, without myrmecophily, habitat specialists, inhabit semi-natural habitat and have sparse colonies. Relative specialists had higher host use in sparse and exposed colonies and on hosts with ant-attendance, compared to relative generalists. Thus, sparse colony aggregation was the only trait that supported both, high average specialization of parasitoid assemblages and high relative specialist advantage.

Dense aphid colonies are more susceptible to generalist predator attacks and spread of fungal infections during humid weather [50]. Thus, specialist parasitoids may avoid intraguild predation, fungal infection and competition by focusing on sparse colonies which are easier for them to locate, compared to generalist parasitoids. These positive effects of focusing on sparse colonies may balance the general disadvantage that they are rare, harder to find and that they represent a lower resource density. Contrary, some generalist parasitoids are shown to have an innate expectancy of a clumped aphid distribution [51] and may leave patches with few aphids more rapidly than when aphids are abundant [52]. Furthermore, specialization might be expected among parasitoids of predictable (more spatio-temporally stable) hosts [11, 53] and population sizes of aphids with dense colonies are shown to be temporally more unstable [50]. Instability of resources may also be an important factor explaining lower average assemblage specialization of parasitoids when using hosts that are either polyphagous, alter plants during their life cycle (heteroecious), habitat generalists or exploit highly dynamic and only temporally available agricultural habitats (related traits, see Fig 3). Movement in space, i.e. switching between plants and habitats can protect aphids from being followed by more specialized parasitoids and they may therefore be more easily parasitized by generalists that are already there [10]. However, feeding on a single plant or in a single habitat does not give any direct protection to aphids and therefore the more specialized species within the parasitoid assemblage may not be more successful in their host use.

Hosts living in non-aggregated, sparse colonies were often highly mobile and without ant-attendance. Mobility is an important trait for aphid defense from parasitoids [10] and highly mobile aphids supported more specialized parasitoids on average, but relative specialists did not have higher host use efficiency. Hence, although highly mobile species are on average attacked more by more specialized parasitoids compared to aphids with low mobility, this does not translate in better host use efficiency of relative specialists. Behavioral defense, such as dropping from the plants increases in adult aphids compared to earlier instars [54], but many parasitoids preferably attack larval stages [55]. It is possible that adults are more often attacked by specialist parasitoids, but there is no difference in specialist/generalist host use efficiency on species with different mobility in our study due to no advantage of specialists when attacking earlier life stages. Similarly, this might be true for aphids with large body sizes as adults of larger aphids can better defend themselves, but earlier, smaller instars are more often parasitized.

Surprisingly, we found higher average parasitoid specialization on aphids that are not ant-attended, although relative specialists species did have advantage on myrmecohilous aphids, especially so on those with facultative myrmecophily. A major benefit for parasitoids attacking ant-attended aphids is avoidance of intraguild predation and hyperparasitism and reduced honeydew in aphid colonies that may promote infection by entomopathogenic fungi [56, 57]. This can increase specialist advantage, compared to relative generalists. On the other hand, the higher mean specialization of parasitoids on aphids that are not ant-attended in our study may result from correlation of this trait with high aphid mobility and low colony aggregation, both of which had higher mean parasitoid specialization. Hence, although parasitoids that are specialized on closely related, ant-attended aphids do have advantage compared to more generalized species, other traits, such as mobility or colony aggregation may have stronger effect on average specialization of the whole parasitoid assemblage. Starý [30] argued that ant-attendance does not influence parasitoid host range, although secondary adaptations and development of defense mechanisms against ant attacks might develop in parasitoids. Aphid behavior and mode of living might instead have a more important role in parasitoid specialization [30]. Additionally, obligate myrmecophiles have higher extinction risks [19] and might be unstable resources for highly specialized parasitoids. For example, *Lysiphlebus cardui* (Marsh.) can reach 100% parasitisation on ant-attended *Aphis fabae cirsiiacanthoidis* leading to eradication of all local aphid colonies [57] and switching to not-attended colonies of this facultative myrmecophile is highly beneficial for parasitoids in such a situation. Furthermore, even if more generalized parasitoids do not seem to be limited by ant-attendance, more specialized species can have an advantage in ant-attended and especially so in facultative ant-attended aphid species that are shown to support larger parasitoid assemblages [19] and therefore exert higher competition pressure.

Hawkins [58, 59] predicted that parasitoid assemblages on concealed hosts should be dominated by generalists whereas those on exposed hosts should be dominated by specialist parasitoids (but see Hrcek et al. [31]). This is because they found idiobionts parasitoids (generalists) to often attack concealed hosts, while koinobionts (specialists) were more common in exposed hosts. In agreement, we found parasitoid specialist advantage of aphid parasitoids (koinobionts) to be higher on exposed hosts. Our finding is largely due to the comparison between exposed hosts and hosts within curled leaves (semi-concealed), since hosts feeding in other feeding niches were rare in our study. Starý [30] showed that the generalist parasitoid *Ephedrus persicae* (Frogatt), which attacks aphids feeding in galls and curled leaves, may attack externally feeding aphids only when their densities are high enough to cause leaves to curl. This may be because curled leaves are highly visible and therefore easily located by generalist parasitoids, while providing minimal physical protection to aphids.

Our study highlights the importance of aphid traits and taxonomic relatedness in parasitoid specialization and host use and contributes to developing a more mechanistic understanding of the species interactions in aphid-parasitoid food webs. It appears that host traits related to local resource characteristics (colony structure, myrmecophily, concealment) are the most important for specialist advantage, while the traits related to host spatial niche and host-plant use were additionally important for mean parasitoid assemblage specialization. Interactions among the host traits as well as other characteristics of aphids (e.g. local abundances, immunological defense), or characteristics of their host-plants (e.g. plant architecture, secondary compounds) not investigated in our study may also be important for parasitoid specialization and host use [13, 14]. Furthermore, since parasitoid host shifts may happen through adaptive mechanisms or through mutual exclusion within local communities [60], the effect of aphid traits on parasitoid host use and specialist advantage remains to be tested in spatio-temporally replicated food webs across different hosts and habitats, as well as experimentally. Importantly, the majority of host traits only had a significant effect on the relative specialist advantage when taxonomic relatedness among hosts was included in the calculation of parasitoid host specificity. Some of these traits showed strong phylogenetic signal (e.g. ant-attendance), supporting evidence that ecologically relevant traits can show phylogenetic conservatism [49], and emphasizing importance of the cost of adaptation on distantly related hosts when comparing parasitoid specialist vs. generalist host use efficiency. These results are in agreement with previous studies that highlighted importance of including host phylogenetic information to assess natural enemy host range in aphid parasitoids [14, 15], fish parasites [61] and bird parasites [62]. In addition to host traits, ability to find and successfully parasitize a host can be under strong selection, thus, parasitoid relatedness can play an important role in determining their food web interactions [62].

## Supporting Information

S1 Table Phylogenetic signal in aphid traits. (DOCX)

S2 Table Aphid × Parasitoid matrix. (CSV)

S3 Table Aphid × Traits matrix. (CSV)

S4 Table Aphid taxonomy. (CSV)
