# Supplementary material for: The Effects of Aphid Traits on Parasitoid Host Use and Specialist Advantage
Source: PLoS One. 2016 Jun 16;11(6):e0157674. doi: 10.1371/journal.pone.0157674 (PMC4910996; doi:10.1371/journal.pone.0157674)
Supplement: S1 Table — (DOCX) [file pone.0157674.s001.docx]

Supplementary information

**Table 1.** Phylogenetic signal in aphid traits. The phylogenetic signal strength in binary traits

(D) was tested running the Fritz and Purvis test with 1,000 permutations (Fritz and Purvis 2010), testing the estimated D value for significant departure from both random association and the clumping expected under a Brownian evolution threshold model, using the R package “caper” (Orme et al. 2013). A D value below 0 reveals a strongly clumped distribution, D ≅ 0 means a “Brownian motion”- like evolutionary distribution, D ≅ 1 a random distribution, and D > 1 an overdispersed distribution. For constructing phylogenetic tree, we used midpoint for rooting and edge lengths of 1 and R packages “picante” (Kembel et al. 2010) and “phangorn” (Schliep 2011).

| Trait | Estimated D | PE(D)random* | PE(D)Brownian** |
| --- | --- | --- | --- |
| Mobility | -0.1787525 | 0 | 0.647 |
| Life cycles | 0.5564546 | 0.002 | 0.116 |
| Colony structure | 0.2521322 | 0.002 | 0.354 |
| Habitat disturbance | 0.7931685 | 0.047 | 0.02 |
| Wax | 0.6676629 | 0.07 | 0.102 |
| Concealment | 1.032299 | 0.527 | 0.002 |
| Habitat specialisation | 0.6049098 | 0.012 | 0.124 |
| Ant-attendance (with vs.without) | -0.2730705 | 0 | 0.695 |
| Body size (small vs. large) | 0.1357992 | 0.004 | 0.506 |
| Feeding (mono- vs. polyphagous) | 0.9762788 | 0.391 | 0.013 |

*Probability of E(D) resulting from no (random) phylogenetic structure

**Probability of E(D) resulting from Brownian phylogenetic structure

References:

David Orme, Rob Freckleton, Gavin Thomas, Thomas Petzoldt, Susanne Fritz, Nick Isaac and Will Pearse (2013). caper: Comparative Analyses of Phylogenetics and Evolution in R. R package version 0.5.2. <http://CRAN.R-project.org/package=caper>

Fritz, S. A. and Purvis, A. (2010). Selectivity in mammalian extinction risk and threat types: a new measure of phylogenetic signal strength in binary traits. Conservation Biology, 24(4):1042-1051.

S.W. Kembel, P.D. Cowan, M.R. Helmus, W.K. Cornwell, H. Morlon, D.D. Ackerly, S.P. Blomberg, and C.O. Webb. 2010. Picante: R tools for integrating phylogenies and ecology. Bioinformatics 26:1463-1464.

Schliep K.P. 2011. phangorn: phylogenetic analysis in R. Bioinformatics, 27(4) 592-593.
